# Spontaneous Epiglottic Hematoma Secondary to Supratherapeutic Anticoagulation

**DOI:** 10.1155/2010/201806

**Published:** 2010-11-07

**Authors:** Cody A. Koch, Steven M. Olsen, Amy M. Saleh, Laura J. Orvidas

**Affiliations:** Department of Otolaryngology-Head and Neck Surgery, Mayo Clinic College of Medicine, Rochester, MN 55905, USA

## Abstract

Hemorrhage into the soft tissues of the airway represents a potentially life-threatening complication of long-term anticoagulation. We report the case of a chronically anticoagulated 37-year-old male who developed a spontaneous hematoma of the epiglottis secondary to a supra-therapeutic INR. Epiglottic hematoma should be considered in the differential of any anticoagulated patient presenting with upper airway compromise. The airway should be secured in a controlled fashion, and the coagulopathy should be rapidly corrected.

## 1. Introduction

Spontaneous hemorrhage frequently complicates anticoagulation with warfarin. An extremely rare but life-threatening complication of anticoagulation with warfarin is hemorrhage into the soft tissues of the airway. We report a case of spontaneous hemorrhage into the epiglottis secondary to supratherapeutic anticoagulation with warfarin.

## 2. Case Report

**Figure 1:**
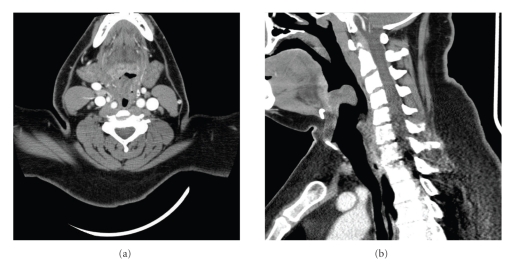
CT scan of the head and neck. (a) Axial and (b) sagittal CT scan images of the neck showing an epiglottic hematoma with moderate narrowing of the airway.

A 37-year-old male with a history of Factor V Leiden mutation receiving chronic anticoagulation with warfarin (15 mg daily) was transferred from an outside emergency department (ED) with the diagnosis of epiglottitis. Four days prior to presentation, the patient experienced left-sided neck pain, odynophagia, and dysphagia but did not seek medical attention. These symptoms resolved; however, approximately 10 hours prior to presentation to the outside ED he experienced acute onset sore throat, right-sided neck pain, odynophagia, and dysphagia. A CT scan of the head and neck (Figure 1) at the outside institution revealed an enlarged epiglottis and was felt to be consistent with epiglottitis. 

Upon arrival at our institution the patient complained of odynophagia, dysphagia, pain with speaking, and right-sided neck pain (6/10) but denied recent illness or fever. On physical examination he was an obese male in no acute distress with stable vital signs (*T* = 36.7°C, HR = 79, BP = 132/65, RR = 23, and SpO2 = 97% on 2 L nasal cannula). He spoke with a muffled voice but did not have audible stridor. Examination of the neck revealed tenderness to palpation on the right side below the submandibular gland. Flexible fiberoptic laryngoscopy revealed a grossly edematous and ecchymotic epiglottis with extension to the right aryepiglottic fold, right false vocal fold, and pyriform sinus. There was also a small isolated area of ecchymosis on the left lateral pharyngeal wall. Laboratory workup revealed anemia (hemoglobin = 9.6 g/dL) with a normal white blood cell count. The patient's INR was elevated at >12.0, which was the maximum limit of detection in our laboratory. The patient admitted that his INR had not been checked in 18 months, and he was taking expired warfarin tablets. 

**Figure 2:**
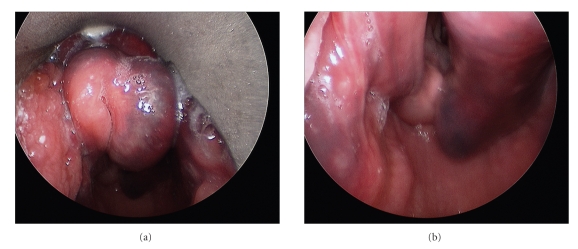
Direct laryngoscopy. (a) Edematous and ecchymotic epiglottis consistent with hematoma as seen on direct laryngoscopy. (b) Extension of the epiglottic hematoma to the right aryepiglottic fold and arytenoid.

Reversal of the patient's elevated INR was immediately started in the ED, and he was admitted to the ICU for observation and normalization of his coagulopathy. Following admission to the ICU the patient developed inspiratory stridor. Given the threat of respiratory compromise an awake tracheostomy was performed without complication. Direct laryngoscopy (Figure 2) confirmed the findings from fiberoptic laryngoscopy. Postoperatively the patient was transferred back to the ICU for further monitoring. 

The remainder of the patient's hospital stay was uncomplicated, and he was discharged home on postoperative day three. The patient was seen in followup seven days later at which time flexible fiberoptic laryngoscopy revealed residual ecchymosis but decreased edema of the airway with no evidence of obstruction. The patient was decannulated and discharged from our care with followup on an as-needed basis. 

## 3. Discussion

Spontaneous hemorrhage as a result of anticoagulation with warfarin is not uncommon; however, spontaneous hemorrhage into the upper airway is rare. Previous reports have described warfarin-associated hemorrhage in the upper airway involving the floor of mouth [1], tongue [2], retropharynx [3], and arytenoids [4]. Lee et al. [5] reported a case of a 41-year-old woman receiving warfarin anticoagulation for a prosthetic mitral valve who developed an acute hematoma of the base of tongue that dissected to involve the epiglottis. We report only the second case of a warfarin-associated hematoma involving the epiglottis and the first case involving the epiglottis primarily, mimicking epiglottitis. 

Warfarin-associated hemorrhage into the upper airway is potentially life threatening and requires an individualized approach to each patient. The patient reported by Lee et al. [5] was able to be managed conservatively with correction of her coagulopathy and inpatient monitoring of her airway. Our patient required more aggressive management (awake tracheostomy) due to a severely supratherapeutic level of anticoagulation, the concern for possible rapid extension of the hematoma and a large consolidated hematoma with anticipated prolonged resolution. 

Regardless of patient presentation, treatment for every patient presenting with warfarin-associated upper airway hemorrhage should include cessation of anticoagulation, rapid correction of the coagulopathy, close airway monitoring in an intensive setting, and a low threshold for securing the airway via tracheostomy. In many cases securing the airway via intubation is not ideal given the anticipated difficulty with both direct and fiberoptic laryngoscopy, the prolonged time course for hematoma resolution, and the potential trauma resulting in additional bleeding in an anti-coagulated patient. Intubation has been used successfully in some reports [1, 6]. 

Hemorrhage of the epiglottis is rare. There are reports of epiglottic hemorrhage arising as a traumatic complication of direct laryngoscopy during endotracheal intubation, as a complication of surgery or trauma [7]. The etiology of this patient's spontaneous upper airway hemorrhage was almost certainly due to the supratherapeutic level of anticoagulation. The incidence of spontaneous hemorrhage in patients taking warfarin is directly related to the level of anticoagulation [8]. Some reports have reported warfarin-associated upper airway hemorrhage following an inciting event such as a severe coughing episode or straining with stooling [6]. Interestingly, no inciting event could be identified in this patient; however, this may have been the second episode of spontaneous upper airway hemorrhage based on history and a separate site of resolving ecchymosis in the left oropharynx on airway evaluation.

## 4. Conclusions

While the incidence of warfarin-associated epiglottic hemorrhage is rare, the life-threatening nature of this complication makes prompt diagnosis and treatment imperative. The possibility of warfarin-associated upper airway hemorrhage should be entertained in any patient presenting with acute onset upper airway obstruction, supratherapeutic INR, and the absence of an elevated white blood cell count and/or fever. The differentiation between epiglottitis and epiglottic hemorrhage, while difficult based on imaging, can easily be made on clinical examination and expeditious treatment initiated.
